# A Computationally Efficient Method for Probabilistic Parameter Threshold Analysis for Health Economic Evaluations

**DOI:** 10.1177/0272989X20937253

**Published:** 2020-07-05

**Authors:** Zoë Pieters, Mark Strong, Virginia E. Pitzer, Philippe Beutels, Joke Bilcke

**Affiliations:** I-BioStat, Data Science Institute, Hasselt University, Hasselt, Limburg, Belgium; Centre for Health Economics Research and Modeling Infectious Diseases (CHERMID), Vaccine and Infectious Disease Institute (VAXINFECTIO), University of Antwerp, Wilrijk, Antwerp, Belgium; School of Health and Related Research (ScHARR), University of Sheffield, Sheffield, UK; Department of Epidemiology of Microbial Diseases, Yale School of Public Health, Yale University, New Haven, CT, USA; Centre for Health Economics Research and Modeling Infectious Diseases (CHERMID), Vaccine and Infectious Disease Institute (VAXINFECTIO), University of Antwerp, Wilrijk, Antwerp, Belgium; Centre for Health Economics Research and Modeling Infectious Diseases (CHERMID), Vaccine and Infectious Disease Institute (VAXINFECTIO), University of Antwerp, Wilrijk, Antwerp, Belgium

**Keywords:** deterministic sensitivity analysis, Monte Carlo approach, probabilistic sensitivity analysis, probabilistic threshold analysis

## Abstract

**Background**. Threshold analysis is used to determine the threshold value of an input parameter at which a health care strategy becomes cost-effective. Typically, it is performed in a deterministic manner, in which inputs are varied one at a time while the remaining inputs are each fixed at their mean value. This approach will result in incorrect threshold values if the cost-effectiveness model is nonlinear or if inputs are correlated. **Objective**. To propose a probabilistic method for performing threshold analysis, which accounts for the joint uncertainty in all input parameters and makes no assumption about the linearity of the cost-effectiveness model. **Methods**. Three methods are compared: 1) deterministic threshold analysis (DTA); 2) a 2-level Monte Carlo approach, which is considered the gold standard; and 3) a regression-based method using a generalized additive model (GAM), which identifies threshold values directly from a probabilistic sensitivity analysis sample. **Results**. We applied the 3 methods to estimate the minimum probability of hospitalization for typhoid fever at which 3 different vaccination strategies become cost-effective in Uganda. The threshold probability of hospitalization at which routine vaccination at 9 months with catchup campaign to 5 years becomes cost-effective is estimated to be 0.060 and 0.061 (95% confidence interval [CI], 0.058–0.064), respectively, for 2-level and GAM. According to DTA, routine vaccination at 9 months with catchup campaign to 5 years would never become cost-effective. The threshold probability at which routine vaccination at 9 months with catchup campaign to 15 years becomes cost-effective is estimated to be 0.092 (DTA), 0.074 (2-level), and 0.072 (95% CI, 0.069–0.075) (GAM). GAM is 430 times faster than the 2-level approach. **Conclusions**. When the cost-effectiveness model is nonlinear, GAM provides similar threshold values to the 2-level Monte Carlo approach and is computationally more efficient. DTA provides incorrect results and should not be used.

## Background

Health economic evaluations compare 2 or more alternative courses of action in terms of costs and consequences. For instance, cost-effectiveness analyses can evaluate if a new health care strategy is preferred over the existing strategy (i.e., considering it *cost-effective*) by comparing costs and health benefits of the strategies.^1^ However, the decisions are often surrounded by considerable uncertainty, which arises from insufficient information about important aspects of the disease process and the different health care strategies under study. The assessment of an uncertain decision involves expressing how confident we are about the best course of action given current information and identifying the most important targets for information gathering through new research.^2^

Several methods have been developed to characterize the sensitivity and uncertainty in health care decisions.^1^ Traditionally, the impact of parameter uncertainty has been explored using a deterministic approach. Input parameter values are varied one at a time, or several at a time, over plausible ranges to test a model outcome’s responsiveness to these variations.^3^ A special case of deterministic sensitivity analysis is deterministic threshold analysis (DTA), which determines the input parameter value at which the preferred health care strategy changes, and is referred to as “the parameter threshold value.”^1,2,4^ Threshold analysis is typically used to determine the price at which a health care strategy becomes cost-effective, but it can also be used, for instance, to determine the minimum disease incidence at which a health care strategy would be cost-effective in a given setting.^5^ Deterministic threshold analysis is straightforward for analysts and easily understood by decision makers. However, one of the major problems with only accounting for uncertainty in a deterministic way is that the estimated cost-effectiveness, and its associated threshold values, can be incorrect in the case of a nonlinear relationship between the input parameters and the model’s outcomes.^1^

Probabilistic sensitivity analysis (PSA) can overcome this limitation.^2,3^ PSA accounts for the plausible values of uncertain input parameters as well as how *likely* each of these values are. The result of a PSA can be used to obtain an unbiased estimate of the expected value of the cost-effectiveness outcome and a quantification of the uncertainty around this outcome. In addition, the relationship between the uncertain input parameters and the corresponding uncertainty around the cost-effectiveness of a health care strategy can be assessed using a range of statistical methods, including value of information analysis.^6^ McCabe et al.^7^ proposed a probabilistic threshold analysis based on a 2-level Monte Carlo approach. In complex health economic evaluations, a 2-level Monte Carlo simulation can be computationally demanding.

We propose an efficient alternative to DTA, namely, a generalized additive model (GAM), that gives correct threshold values in the case of a nonlinear relationship between inputs and outputs of the health economic model while accounting for the uncertainty in all other input parameters. We evaluate the accuracy and computational efficiency of GAM in estimating threshold values by comparing it with the 2-level Monte Carlo probabilistic threshold analysis. Our working example is a recent peer-reviewed health economic evaluation of vaccination against typhoid fever. In this example, there is a nonlinear relationship between the uncertain input parameters and the corresponding cost-effectiveness of the typhoid vaccination program, which is due in part to the use of a dynamic transmission model.^8^

## Methods

### Net Benefit as Measure for Cost-Effectiveness

A health economic evaluation compares the costs and health effects (such as deaths or disability-adjusted life-years [DALYs] averted) of alternative courses of action (including “current practice”). As such, it informs decision makers about the relative efficiency of a change in policy (e.g., the adoption of a new policy option). The relative efficiency of one policy option v. another is usually expressed as an incremental cost-effectiveness ratio (ICER) or as an incremental net monetary (or health) benefit. Throughout this article, we use incremental net monetary benefit (INB) as the measure of cost-effectiveness so that the threshold methods we propose are general applicable. Indeed, when uncertainty is accounted for in a probabilistic way, the expected ICER is only interpretable when comparing 2 decision options (e.g., new strategy v. current strategy) and when all incremental costs and effects are positive.^9^ The INB is defined as

(1) $$\begin{array}{cc} {\mathrm{INB}}_{d} & =\lambda \Delta E_{d}-\Delta C_{d}\\ & =\lambda (E_{d}-E_{d_{0}})-(C_{d}-C_{d_{0}}) \end{array}$$

where ${\mathrm{INB}}_{d}$ represents the incremental net benefit for option d(d=1,…,D), one of D alternative health care strategies under consideration, relative to the baseline strategy, $d_{0}$; λ represents the decision maker’s maximum willingness to pay (WTP) per unit gain in health; $\Delta E_{d}$ is the incremental health benefit of option d compared to the baseline strategy; and $\Delta C_{d}$ is the difference in costs between strategy d and the baseline strategy. The optimal health care strategy is that which has the highest INB.^1,9,10^ The current strategy is typically chosen as the baseline strategy, but any strategy can be chosen, as long as the same baseline strategy is chosen for all options compared.

Usually, there is considerable uncertainty around the expected values of input parameters due to limited evidence on the expected costs and effects of a health care strategy d. Therefore, the expected ${\mathrm{INB}}_{d}$ will also be surrounded with uncertainty. We account for this uncertainty by assigning appropriate probability distributions to the input parameters (θ), denoted by p(θ).^1^ We sample K values from p(θ) and calculate the K corresponding ${\mathrm{INB}}_{d}$ values. The most cost-effective strategy is the strategy with the highest expected incremental net benefit. We adapt equation (1):

(2) $$E_{\theta }[\;INB{\;}_{d}(\theta )]≃\frac{1}{K}\sum_{k=1}^{K}\left\{\lambda (E_{d}^{\left(k\right)}-E_{d_{0}}^{\left(k\right)})-(C_{d}^{\left(k\right)}-C_{d_{0}}^{\left(k\right)})\right\},$$

where $E_{\theta }[{\mathrm{INB}}_{d}(\theta )]$ denotes the expected INB of health care strategy d based on distribution of all parameters.

### Definition of Threshold Value

The threshold value for a parameter $\theta_{i}$ is the value $\theta_{i}^{*}$, for which the following 2 conditions hold:

1. We can identify decision options, d′ and d″, where d′≠d″, which have expected net benefits, conditional on $\theta_{i}$, that are equal, that is,

(3) $$E_{\theta_{-i}|\theta_{i}}[{\mathrm{INB}}_{d\prime }(\theta_{i},\theta_{-i})]=E_{\theta_{-i}|\theta_{i}}[{\mathrm{INB}}_{d\prime \prime }(\theta_{i},\theta_{-i})].$$

2. There must be no decision option with expected net benefit, conditional on $\theta_{i}$, that is greater than that for d′, that is,

(4) $$E_{\theta_{-i}|\theta_{i}}[{\mathrm{INB}}_{d\prime }(\theta_{i},\theta_{-i})]\geq E_{\theta_{-i}|\theta_{i}}[{\mathrm{INB}}_{d}(\theta_{i},\theta_{-i})]$$

for all d,

where $\theta_{i}$ in equations (3) and (4) has an appropriate probability distribution ($p(\theta_{i})$) to characterize its uncertainty. The first condition determines that $\theta_{i}^{*}$ is a value for which decision option d′ has the same conditional expected net benefit as option d″ and is therefore a *threshold* value, and the second condition determines that $\theta_{i}^{*}$ is a threshold value where the *optimal* health care strategy changes. Determining the threshold value, $\theta_{i}^{*}$, in the net benefit framework leads to a dependence between $\theta_{i}^{*}$ and the chosen WTP value, λ.

### Deterministic Threshold Analysis

Deterministic threshold analysis seeks to identify the value of a parameter for which the optimal health care strategy changes while keeping all other input parameters constant. More formally, the deterministic parameter threshold value ($\theta_{i}^{*}$) must satisfy 2 conditions:

1. $\theta_{i}$ is the parameter for which we can identify policy options, d′ and d″, where d′≠d″, which have INB, conditional on $\theta_{i}$, that are equal, that is,

(5) $${\mathrm{INB}}_{d\prime }(\theta_{i},E[\theta_{-i}])=\;{\mathrm{INB}}_{d\prime \prime }(\theta_{i},E[\theta_{-i}]).$$

2. There must be no decision option with net benefit (conditional on $\theta_{i}$) greater than that for d′, that is,

(6) $${\mathrm{INB}}_{d\prime }(\theta_{i},E[\theta_{-i}])\geq \;\mathrm{IN}B_{d\prime \prime }(\theta_{i},E[\theta_{-i}])$$

for all d,

where $\theta_{i}$ in equations (5) and (6) refers to point estimates. The first condition determines that $\theta_{i}^{*}$ is a parameter value where decision option d′ has the same INB (evaluated at the mean values of $\theta_{-i}$) as option d″, and the second condition determines that $\theta_{i}^{*}$ is a threshold value, where the *optimal* health care strategy changes to d′. Again, the threshold value, $\theta_{i}^{*}$, depends on the chosen WTP value, λ, in the net benefit framework.

The analysis proceeds as follows^11,12^:

1. Define the uncertain parameter of interest, $\theta_{i}$.
2. Fix remaining input parameters $\theta_{-i}(i\neq -i)$ at their expected values.
3. The threshold value $\theta_{i}^{*}$ can be obtained in the following ways:
 (a) Graphically: Vary the values $\theta_{i}$ (generally 5–10 different values) and assess the impact on the cost-effectiveness (e.g., plot ${\mathrm{INB}}_{d}$ for each health care strategy d relative to a baseline option d=0 as a function of the different values of the uncertain parameter of interest); the point at which any of the top 2 lines cross is $\theta_{i}^{*}$.
 (b) Algebraically: Solve the linear system composed of 2 health economic models, one for d′ and the other for d″, to find $\theta_{i}^{*}$ satisfying equations (5) and (6). Solve the linear system for any combination of d′ and d″. Make sure that $\theta_{i}^{*}$ is obtained within the range of plausible values of $\theta_{i}$.
 (c) Numerically:
  - Vary over K (k=1,…,K) values of $\theta_{i}$ and record the corresponding ${\mathrm{INB}}_{d}$, for each d.
  - Sort the values of ${\mathrm{INB}}_{d}$, for each d, according to ascending $\theta_{i}$ values.
  - Set ${\mathrm{INB}}_{d}^{\left(k\right)}=0$ for the “baseline” decision option *d* = 0.
  - Determine the health care strategy with the highest ${\mathrm{INB}}_{d}$ for $\theta_{i}^{\left(k\right)}$, $d^{\left(k\right)}=\arg\max_{d}{\mathrm{INB}}_{d}^{\left(k\right)}$.
  - Determine any value, $k^{*}$, such that $d^{\left(k^{*}\right)}\neq d^{\left(k^{*}+1\right)}$.
  - Each $k^{*}$ will define a threshold value $\theta_{i}^{*}$ that lies in the interval $\theta_{i}^{\left(k^{*}\right)}<\theta_{i}^{*}<\theta_{i}^{\left(k^{*}+1\right)}$ (see note).
**Note:** There may be no values of $k^{*}$, in which case, there are no threshold values, and the optimal health care strategy does not depend on the value of the input parameter $\theta_{i}$ considered. Or there may be a single value of $k^{*}$, in which case there is a single threshold value, $\theta_{i}^{*}$. Since the closest we can get to $\theta_{i}^{*}$ is $\theta_{i}^{\left(k^{*}\right)}$ or $\theta_{i}^{\left(k^{*}+1\right)}$, we approximate $\theta_{i}^{*}$ by the midpoint of the interval.

If the cost-effectiveness measure (${\mathrm{INB}}_{d}$) has a nonlinear relationship with the input parameters $\theta_{-i}$, then

(7) $$E_{\theta_{-i}|\theta_{i}}[{\mathrm{INB}}_{d}(\theta_{i},\theta_{-i})]\neq {\mathrm{INB}}_{d}(\theta_{i},E[\theta_{-i}]).$$

Consequently, a deterministic threshold analysis will result in an incorrect estimate of the threshold value $\theta_{i}^{*}$.^13^

### Probabilistic Parameter Threshold Analysis

When a deterministic threshold analysis results in incorrect estimates for $\theta_{i}^{*}$, one can rely on a probabilistic parameter threshold analysis. The advantage of a probabilistic parameter threshold analysis is that it incorporates the joint uncertainty in all parameters, resulting in the correct estimation of $\theta_{i}^{*}$ even when nonlinear relationships exist between ${\mathrm{INB}}_{d}$ and $\theta_{-i}$. The key to finding the value $\theta_{i}^{*}$ that satisfies the conditions (3) and (4) lies in finding a way to estimate $E_{\theta_{-i}|\theta_{i}}[{\mathrm{INB}}_{d}(\theta_{i},\theta_{-i})]$. The most obvious way to do this is via Monte Carlo sampling, but this leads to a “nested” 2-level scheme in which values of $\theta_{i}$ are sampled in an outer loop, and conditional on this, values of $\theta_{-i}$ are sampled in an inner loop. The existing 2-level Monte Carlo approach is computationally costly; therefore, we propose an alternative method to estimate $E_{\theta_{-i}|\theta_{i}}[{\mathrm{INB}}_{d}(\theta_{i},\theta_{-i})]$ using a nonparametric regression-based method, called a GAM, first proposed by Strong et al.^14^

### Two-Level Monte Carlo Approach

We can estimate the term $E_{\theta_{-i}|\theta_{i}}[{\mathrm{INB}}_{d}(\theta_{i},\theta_{-i})]$ in equations (3) and (4) using a 2-level Monte Carlo approach. A detailed overview of the approach is given in Algorithm 1.

**Algorithm 1:** Two-Level Monte Carlo Scheme for Estimating Threshold Value $\theta_{i}^{*}$ for Parameter $\theta_{i}$

**1** Sample K times from the distribution of the parameter of interest $p(\theta_{i})$.
**2** Order sampled values such that $\theta_{i}^{\left(1\right)}<\theta_{i}^{\left(2\right)}<\ldots <\theta_{i}^{\left(K-1\right)}<\theta_{i}^{\left(K\right)}$.
**3 for** k=1 **to** K **do**
**4 for** j=1 **to** J **do**
**5** Sample $\theta_{-i}^{\left(j,k\right)}$ from the conditional distribution of the remaining parameters, $p(\theta_{-i}|\theta_{i}^{\left(k\right)})$(the same parameter uncertainty distributions are assumed as in PSA).
**6** Evaluate the incremental net benefit function for each d and store ${\mathrm{INB}}_{d}(\theta_{i}^{\left(k\right)},\theta_{-i}^{\left(j,k\right)})$.
**7 end**
**8** Compute and store inner loop mean for each of the alternative strategies d=1,…,D, ${\bar{\mathrm{INB}}}_{d}^{\left(k\right)}=\frac{1}{J}\sum_{j=1}^{J}{\mathrm{INB}}_{d}(\theta_{i}^{\left(k\right)},\theta_{-i}^{\left(j,k\right)})$. These are estimates of the conditional expected value $E_{\theta_{-i}|\theta_{i}}[{\mathrm{INB}}_{d}(\theta_{i}^{\left(k\right)},\theta_{-i})]$.
**9** Set ${\bar{\mathrm{INB}}}_{d}^{\left(k\right)}=0$ for the “baseline” decision option d=0.
**10** Determine the policy option with the highest expected INB given $\theta_{i}^{\left(k\right)}$, $d^{\left(k\right)}=\arg\;\max_{d}{\bar{\mathrm{INB}}}_{d}^{\left(k\right)}$.
**11 end**
**12** Determine any value(s), $k^{*}$, such that $d^{\left(k^{*}\right)}\neq d^{\left(k^{*}+1\right)}$ (see note).
**13** Each $k^{*}$ will define a threshold value $\theta_{i}^{*}$ that lies in the interval $\theta_{i}^{\left(k^{*}\right)}<\theta_{i}^{*}<\theta_{i}^{\left(k^{*}+1\right)}$.

**Note:** There may be no values of $k^{*}$, in which case there are no threshold values, and the optimal health care strategy does not depend on the value of the input parameter $\theta_{i}$ considered. There may be a single value of $k^{*}$, in which case, there is a single threshold value, $\theta_{i}^{*}$. Or, there may be multiple values of $k^{*}$ and therefore multiple threshold values. We approximate $\theta_{i}^{*}$ by the midpoint of the interval $\left\{\theta_{i}^{\left(k^{*}\right)},\theta_{i}^{\left(k^{*}+1\right)}\right\}$. This is justified as long as sufficient values are sampled from the distribution of $\theta_{i}$.

This approach is very computationally intensive for all models except for very simple models due to the need to evaluate the incremental net benefit function K×J times.^14–16^ Ideally, K should span the range of the parameter of interest $\theta_{i}$, and K and J should be large. In practice, a stepwise approach can be used to determine the area of the input parameter (outer loop) containing the threshold value. Indeed, due to the complexity of the chosen health economic evaluation (section “A Real-World Example”), we were only able to sample 7 values from $p(\theta_{i})$ (K=7) in order to keep J large (J=10,000). At first, we used a broad range of $\theta_{i}$ and narrowed the range until we had a precise (up to 3 decimals) parameter threshold value. Since we performed only a limited number of outer iterations, we refer to this approach as the adjusted 2-level Monte Carlo approach. Last, we recommend the values of J be varied until a stable $\theta_{i}^{*}$ is obtained.

### Regression-Based Approach Using a Generalized Additive Model

As an alternative to the 2-level Monte Carlo approach, we propose a meta-model approach, based on a GAM, summarizing the relationship between the inputs and the outputs postsimulation. This regression-based approach only requires the PSA sample to correctly estimate $\theta_{i}^{*}$ while satisfying the conditions in (3) and (4).

A GAM allows for flexible specification of the relationship between the ${\mathrm{INB}}_{d}$ and the input parameters θ for each health care strategy under consideration. Hence, detailed parametric specifications are not needed. First, we define the PSA sample as a set of K samples from the joint distribution of the model input parameters, $\left\{\theta^{1},\ldots ,\theta^{K}\right\}$, and the corresponding evaluations of the INB function $\left\{{\mathrm{INB}}_{d}(\theta^{1}),\ldots ,{\mathrm{INB}}_{d}(\theta^{K})\right\}$ for each health care strategy d=1,…,D compared to a baseline option $d_{0}$. In general, a GAM is defined as follows:

(8) $${\mathrm{INB}}_{d}(\theta_{i}^{k})=E_{\theta_{-i}|\theta_{i}^{k}}[{\mathrm{INB}}_{d}(\theta_{i}^{k},\theta_{-i})]+\epsilon^{k}$$

(9) $$=g_{d}(\theta_{i}^{k})+\epsilon^{k},$$

where equation (8) expresses the ${\mathrm{INB}}_{d}$ as the sum of the conditional expectation we require and a mean-zero error term ϵ, and equation (9) reexpresses the conditional expectation as an unknown function of $\theta_{i}$. See Strong et al.^14^ for a detailed derivation.

We do not know the form of the unknown function $g_{d}(\theta_{i})$, but we do expect it to be smooth, so we choose to model it using a GAM. Different choices can be made for the smooth function s(·) (equation (10)), but a typical choice is a third-order polynomial spline. A third-order polynomial spline is a curve constructed from sections of cubic polynomials that are joined together end to end at a series of “knots.” Any cubic spline can also be represented by the weighted sum of a series of “basis” functions (in the same way that any sound wave can be constructed from the sum of a series of sine waves of different frequencies) Thus, we can write

(10) $$g_{d}(\theta_{i})=s(\theta_{i})=\sum_{l=1}^{L}\beta_{l}b_{l}(\theta_{i}),$$

where $b_{l}(\cdot )$ are basis functions, with corresponding weights $\beta_{l}$ that are estimated from the data. The value L and smoothing parameter control the model’s smoothness. The latter adds a penalty to the likelihood of the spline to suppress overly flexible terms. In the implementation of the GAM in the mgcv package in R, the optimal penalty is by default learned from the data using cross-validation, while the value L must be prechosen and is fixed to be large.^17^ In our example, we chose cubic regression splines with dimension 20 and smoothing parameter obtained using cross-validation to model the data. We obtained the basis and the dimension after a sensitivity analysis. Changing both the basis and the dimension did not influence the threshold value. Therefore, we opted for a combination of basis and dimension that provided a stable threshold value and was not too computationally demanding at the same time (for a detailed overview, see Appendix C, available online). For a more extended explanation on GAMs, we refer to other sources.^14,17^

We propose algorithm 2 to obtain the parameter threshold value, $\theta_{i}^{*}$, using a GAM.

**Algorithm 2:** Regression-Based Scheme for Estimating Threshold Value $\theta_{i}^{*}$ for Parameter $\theta_{i}$

**1** Sample K times from the joint distribution of all parameters p(θ).
**2** Order sampled values of θ with respect to $\theta_{i}$ such that $\theta_{i}^{\left(1\right)}<\theta_{i}^{\left(2\right)}<\ldots <\theta_{i}^{\left(K-1\right)}<\theta_{i}^{\left(K\right)}$.
**3 for** k=1 **to** K **do**
**4** Evaluate the incremental net benefit function for each d and store ${\mathrm{INB}}_{d}(\theta^{\left(k\right)})$. This is the standard “PSA” sample.
**5 end**
**6 for** d=1 **to** D **do**
**7** Regress ${\mathrm{INB}}_{d}(\theta^{\left(1,\ldots ,K\right)})$ on $\theta_{i}^{\left(1,\ldots ,K\right)}$ using a GAM (R code available in Appendix A, available online).
**8** Compute the regression fitted values, ${\hat{\mathrm{INB}}}_{d}^{\left(1,\;\ldots \;,\;K\right)}$. These are estimates of the conditional expected values $E_{\theta_{-i}|\theta_{i}}[{\mathrm{INB}}_{d}(\theta_{i}^{\left(1,\;\ldots \;,K\right)},\theta_{-i})]$.
**9 end**
**10 for** k=1 **to** K **do**
**11** Set ${\hat{\mathrm{INB}}}_{d}^{\left(k\right)}=0$ for the “baseline” decision option d=0.
**12** Determine the policy option with the highest expected INB given $\theta_{i}^{\left(k\right)}$, $d^{\left(k\right)}=\arg\max_{d}{\hat{\mathrm{INB}}}_{d}^{\left(k\right)}$.
**13 end**
**14** Determine any value(s), $k^{*}$, such that $d^{\left(k^{*}\right)}\neq d^{\left(k^{*}+1\right)}$ (see note).
**15** Each $k^{*}$ will define a threshold value $\theta_{i}^{*}$ that lies in the interval $\theta_{i}^{\left(k^{*}\right)}<\theta_{i}^{*}<\theta_{i}^{\left(k^{*}+1\right)}$ (A function, written in R,^18^ is available in Appendix A, available online).

**Note:** There may be no values of $k^{*}$, in which case, there are no threshold values. There may be a single value of $k^{*}$, in which case, there is a single threshold value, $\theta_{i}^{*}$. Or, there may be multiple values of $k^{*}$ and therefore multiple threshold values. We approximate $\theta_{i}^{*}$ by the midpoint of the interval $\left\{\theta_{i}^{\left(k^{*}\right)},\theta_{i}^{\left(k^{*}+1\right)}\right\}$.

### Quantification of Uncertainty

We use a bootstrap procedure to provide a measure of precision and accuracy of the parameter threshold value in the presence of possible model violations. We opted for a nonparametric bootstrap because it does not rely on asymptotic normality and hence will be applicable for a wider range of applications. If asymptotic normality holds, Strong et al.^14^ described a method to obtain the standard errors directly from the GAM. The nonparametric bootstrap relies on sampling with replacement from the observed PSA sample {θ,INB}. We sample B times from the PSA sample, generating b=1,…,B bootstrap sampled versions of the PSA sample $\left\{\theta^{b},\mathrm{IN}B^{b}\right\}$. For each of the bootstrap samples, the parameter threshold value, $\theta^{*b}$, is calculated using algorithm 2. The uncertainty about the threshold value can then be expressed through a (1−α)% interval from $\theta_{\left(\alpha /2\right)}^{*}$ to $\theta_{\left(1-\alpha /2\right)}^{*}$ where $\theta_{\left(\alpha /2\right)}^{*}$ represents the α/2 percentile of the bootstrap values $\theta^{*b}$.^19,20^

Depending on the bootstrap sample, a different number of parameter threshold values might arise compared to the original PSA sample, particularly when the input parameter does not influence the cost-effective strategy (i.e., low expected value of obtaining perfect information [EVPPI] value). To acknowledge this type of uncertainty about the number of threshold values, we denoted the number of bootstrap samples resulting in the same number of threshold values as the original PSA sample as $B_{\mathrm{retain}}$. The lower $B_{\mathrm{retain}}$, the more uncertainty there is about the number of threshold values. If a bootstrap sample produces a different number of threshold values than the original PSA sample, then this bootstrap sample is discarded before calculating the bootstrap uncertainty interval.

### A Real-World Example

We chose a health economic evaluation comparing typhoid conjugate vaccination strategies in Gavi-eligible countries as a real-world example, in which there is a nonlinear and even nonmonotone relationship between some of the uncertain input parameters and the corresponding cost-effectiveness of the typhoid vaccination program. This example allowed us to illustrate the various possible outcomes of threshold analysis. The health economic evaluation aimed to inform decision makers on the cost-effectiveness of 3 different vaccination strategies compared to each other and to no vaccination (no vac; $d_{0}$): routine vaccination of infants at 9 months of age or routine vaccination at 9 months with a catchup campaign up to either 5 years (RC5) or 15 years of age (RC15). In this article, threshold values were determined for an evaluation comparing only 2 health care strategies (vaccination strategy RC15 compared to the baseline option [no vac]) and for an evaluation comparing 3 health care strategies (vaccination strategies RC5 and RC15 v. the baseline option [no vac]), since routine vaccination without catchup was never the optimal strategy in the original analysis.^8^

We chose to obtain threshold values for uncertain input parameters for 3 countries (Nicaragua, Uganda, and Cambodia), assuming WTP values per DALY averted that allowed us to illustrate different possible outcomes (no threshold value, a single threshold value, and more than 1 threshold value).

We assessed parameter threshold values for 3 uncertain input parameters: typhoid case fatality risk when hospitalized ($\mathrm{CF}R_{\mathrm{hosp}}$), the probability of hospitalization for typhoid fever (Pr(hosp)), and the duration of illness for patients seeking medical care ($\mathrm{DO}I_{\mathrm{care}}$) (Table 1). The case fatality risk and probability of hospitalization were chosen because in some settings, they had a nonlinear, respectively, nonmonotone relationship with the cost-effectiveness outcome and had a big impact on the optimal health care strategy (i.e., they had the highest EVPPI), for the countries and WTP values we considered. Hence, threshold values for these parameters informed changes to the optimal strategy. The parameter duration of illness was chosen because it had a much lower EVPPI value for the countries and WTP values considered and hence less impact on the optimal strategy. This parameter was chosen to illustrate the performance of the different threshold methods when a threshold value was not necessarily expected.

**Table 1:** Distributional Characteristics of the Uncertain Input Parameters

Parameter	Mean	Median	95% Credible Interval	Uncertainty Distribution
$\mathrm{CF}R_{\mathrm{hosp}}$	0.059	0.044	0.008–0.196	$\mathrm{logi}t^{-1}(N(-3.07,0.87)$
Pr(hosp)	0.061	0.038	0.004–0.249	$\mathrm{logi}t^{-1}(N(-3.25,1.20)$
$\mathrm{DO}I_{\mathrm{care}}$ (years)	0.043	0.043	0.034–0.054	Gamma(16,2)/365

Table 1 shows the uncertainty distributions for the 3 input parameters considered in this article. The uncertainty distributions around the expected case fatality risk and probability for hospitalization are right skewed. Their means and standard errors are estimated from a random-effects meta-analysis. As a consequence of the logistic regression model, the standard errors are only available on the logit scale. After sampling from the normal distribution on the logit scale, the values are transformed to their original scale using the inverse logit ($\frac{e^{x}}{1+e^{x}}$). The mean and standard error of $\mathrm{DO}I_{\mathrm{care}}$ are also estimated using a random-effects meta-analysis. Since $\mathrm{DO}I_{\mathrm{care}}$ is Poisson distributed, we sample from a Gamma distribution. The sampled values are rescaled such that $\mathrm{DO}I_{\mathrm{care}}$ is expressed in years.^8^

## Results

**Figure 1:**
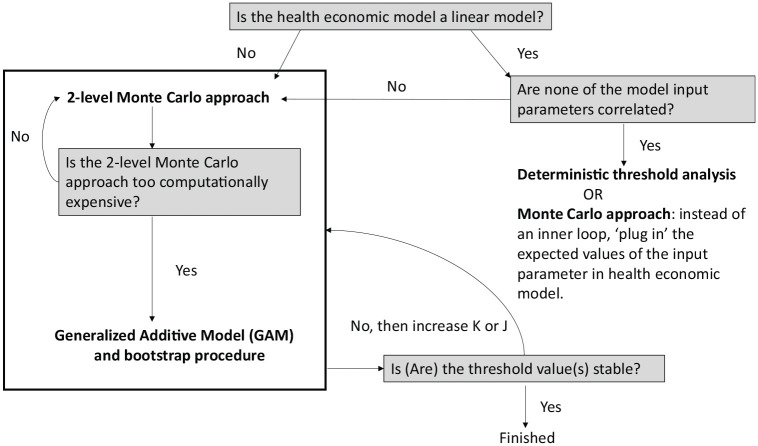
A guide for performing parameter threshold analysis.

The appropriate method to perform threshold analysis depends on the features of the health economic model. Figure 1 presents a flowchart describing the most suitable method to carry out parameter threshold analysis. A GAM would be the most suitable method to obtain parameter threshold values in our example due to the nonlinear relationship between the uncertain input parameters and ${\mathrm{INB}}_{d}$. However, we perform all 3 methods—a deterministic threshold analysis, an adjusted 2-level Monte Carlo method, and a GAM—to compare the threshold value(s) obtained by each method. Table 2 shows the parameter threshold values for different scenarios. We kept the size of the samples equal in all scenarios and for all input parameters (K=10,000 for GAM and deterministic threshold analysis, K=7 and J=10,000 for the adjusted 2-level Monte Carlo analysis). The same holds for the number of values for K that were used in the adjusted 2-level Monte Carlo approach (K=7) (Appendix B, available online).

**Table 2:** Comparison of the Parameter Threshold Values Obtained with DTA, Adjusted 2-Level MC Approach, and GAM for Different Settings^a^

		DTA			Adjusted 2-Level MC			GAM					
		K=10,000			K=7, J=10,000			Cubic Regression Splines, L=20, K=10,000					
$\theta_{i}$	EVPPI	$\theta_{i}^{*}$	$d^{\left(k^{*}+1\right)}$	Time^b^	$\theta_{i}^{*}$	$d^{\left(k^{*}+1\right)}$	Time^b^	$\theta_{i}^{*}$	$d^{\left(k^{*}+1\right)}$	Time^b^	95% CI	$B_{\mathrm{retain}}/B$	Time^b^
**Nicaragua, WTP = $1000, 1 health care strategy (D=1; RC15) compared to no vaccination ($d_{0}$)**													
$\mathrm{CF}R_{\mathrm{hosp}}$	700,094	0.062	RC15	16.6	0.040	RC15	235.6	0.036	RC15	0.6	0.035–0.039	998/1000	159.5
Pr(hosp)	1,276,475	0.052	RC15	14.4	0.043	RC15	220.0	0.041	RC15	0.6	0.040–0.043	1000/1000	160.2
$DOI_{\mathrm{care}}$	0	None^c^	No vac	14.6	None	RC15	231.0	None	RC15	0.5	NA	391/1000	159.2
**Nicaragua, WTP = $1000, 2 health care strategies (D=2; RC5 and RC15) compared to no vaccination ($d_{0}$)**													
$\mathrm{CF}R_{\mathrm{hosp}}$	1,860,599	0.113	RC15	15.9	0.069	RC15	219.2	0.064	RC15	0.7	0.060–0.071	983/1000	199.1
Pr(hosp)	2,665,148	0.093	RC15	14.5	0.074	RC15	207.7	0.070	RC15	0.6	0.066–0.073	1000/1000	189.9
$DOI_{\mathrm{care}}$	0	None	No vac	16.7	None	No vac	214.3	None	No vac	0.6	NA	191/1000	189.6
**Uganda, WTP = $800, 2 health care strategies (D=2; RC5 and RC15) compared to no vaccination ($d_{0}$)**													
$\mathrm{CF}R_{\mathrm{hosp}}$	18,451,230	0.109	RC15	23.3	0.056 0.070	RC5 RC15	241.0	0.058 0.071	RC5 RC15	0.6	0.052–0.061 0.065–0.076	965/1000	199.2
Pr(hosp)	25,018,120	0.092	RC15	14.9	0.060 0.074	RC5 RC15	214.8	0.061 0.072	RC5 RC15	0.6	0.058–0.064 0.069–0.075	999/1000	194.5
$DOI_{\mathrm{care}}$	4256	None	No vac	14.9	None	RC5	217.3	0.033	RC5	0.7	0.030–0.061	98/1000	202.1
**Cambodia, WTP = $100, 2 health care strategies (D=2; RC5 and RC15) compared to no vaccination ($d_{0}$)**													
$\mathrm{CF}R_{\mathrm{hosp}}$	2,788,364	0.114	RC15	16.8	0.053 0.080	RC5 RC15	227.5	0.055 0.083	RC5 RC15	0.6	0.052–0.062 0.076–0.091	955/1000	188.9
Pr(hosp)	4,837,512	0.083	RC15	16.5	0.059 0.083	RC5 RC15	226.8	0.060 0.085	RC5 RC15	0.7	0.058–0.062 0.081–0.090	998/1000	191.4
**Cambodia, WTP = $8000, 2 health care strategies (D=2; RC5 and RC15) compared to no vaccination ($d_{0}$)**													
$\mathrm{CF}R_{\mathrm{hosp}}$	0	None	RC15	13.7	None	RC15	219.1	None	RC15	0.6	NA	966/1000	188.5
Pr(hosp)	0	None	RC15	14.5	None	RC15	218.6	None	RC15	0.6	NA	714/1000	198.4

CI, confidence interval; DTA, deterministic threshold analysis; EVPPI, expected value of partial perfect information; GAM, generalized additive model; MC, Monte Carlo; NA, not applicable when no parameter threshold value is obtained; No vac, no vaccination; RC5, routine vaccination with catchup campaign to 5 years; RC15, routine vaccination with catchup campaign up 15 years; WTP = willingness to pay for 1 disability-adjusted life-year averted (in USD).

a $\theta_{i}=$ parameter of interest; $\theta_{i}^{*}=$ threshold value(s), if present, for $\theta_{i}$; $d^{\left(k^{*}+1\right)}=$ health care strategy with the highest expected incremental net monetary benefit (INB) at $\theta_{i}^{\left(k^{*}+1\right)}$. If not mentioned otherwise, the health care strategy at $d^{k^{*}}$ is no vaccination; $B_{\mathrm{retain}}$ = number of bootstrap samples retained to calculate the 95% CI; EVPPI quantifies the value of obtaining perfect information on the parameter of interest. The EVPPI is calculated based on Strong et al.^14^

b Indicate the time needed to perform, respectively, the method and the bootstrap (GAM: excluding the time needed to obtain the probabilistic sensitivity analysis sample).

c “None” indicates that no parameter threshold value was obtained, meaning that the health care strategy with the highest expected INB remains the same and is denoted under $d^{\left(k^{*}+1\right)}$.

Deterministic threshold analysis is computationally faster than the adjusted 2-level Monte Carlo approach but slower than GAM. In this example, it consistently overestimates the value of $\theta_{i}^{*}$ (i.e., it overestimates the minimum value at which a vaccination strategy is preferred over no vaccination). Where the adjusted 2-level Monte Carlo approach and GAM result in 2 parameter threshold values, deterministic threshold analysis is only able to obtain 1. According to the deterministic threshold analysis, RC5 will never be the optimal health care strategy.

GAM is able to calculate the threshold value(s) in a fraction of the time that is needed for the adjusted 2-level Monte Carlo approach. Although the bootstrap procedure is time-consuming, GAM is still faster than the adjusted 2-level Monte Carlo approach.

There is a good agreement between GAM and the adjusted 2-level Monte Carlo approach. In most settings, the 2 approaches provide a parameter threshold value that is precise up to 2 decimals, with the exception of the input parameter $\mathrm{DO}I_{\mathrm{care}}$ in Uganda. In Uganda (WTP = $800, D=2), the adjusted 2-level Monte Carlo approach shows no parameter threshold value, whereas GAM does suggest a threshold value; however, the 95% CI of the threshold $\mathrm{DO}I_{\mathrm{care}}$ in the GAM approach spans almost the entire range of possible values for that parameter, indicating a lot of uncertainty about the parameter threshold value. The proportion of bootstrap samples retained was low for $\mathrm{DO}I_{\mathrm{care}}$ in Nicaragua and Uganda, indicating uncertainty about whether and how many threshold values could be identified. Therefore, we do not recommend to interpret threshold values when the number of bootstrap samples retained is low. For more technical details, see Appendix D (available online).

For Cambodia, we considered 2 different WTP values, $100 and $8000. When we considered a WTP value of $100, both the adjusted 2-level Monte Carlo approach and GAM find 2 threshold values. For the higher WTP value, no parameter threshold values are found. This was expected, since the EVPPI was low at the higher WTP value.

## Discussion

We propose GAM as a novel regression-based approach to calculate a parameter’s threshold value(s) in health economic evaluations. The GAM approach only requires the PSA sample of a cost-effectiveness analysis and is flexible, easy to use, and computationally efficient. In our example, GAM does not provide incorrect threshold values or fails to find threshold values (as the deterministic approach does). GAM also outperforms the 2-level Monte Carlo approach in terms of computational time.

GAM has several advantages over the existing methods. First, GAM results in the same threshold values as the adjusted 2-level Monte Carlo approach when cost-effectiveness measures are nonlinearly related to the inputs, unlike the deterministic threshold analysis. Our example (Table 2) showed that threshold values were overestimated and that not all threshold values were identified with the deterministic threshold approach. Therefore, threshold values obtained from a deterministic threshold analysis should not be interpreted when there is a nonlinear relationship between inputs and outputs. Second, GAM is easy to use because it relies on the PSA sample to account for uncertainty in the input parameters’ distribution, and there is no need to assume plausible values as in the deterministic threshold approach.^21^ Third, GAM is computationally fast compared to the 2-level Monte Carlo approach. In order to perform the 2-level Monte Carlo approach, we needed at least 208 seconds for K=7. The time needed to perform a GAM, including the bootstrap procedure, was at most 199 seconds (Table 2). Last, threshold values obtained by GAM were quite robust against changes in dimension and the smoothing function chosen (Appendix C, available online).

There are some limitations of this work. First, we performed the comparison of the 3 threshold approaches on only 1 example. However, this proved to be sufficient to show the incorrectness in the deterministic threshold values. Second, we were limited in the number of samples we could use in the 2-level Monte Carlo approach because running our health economic evaluation was computationally too intensive. Thus, we could not perform a complete 2-level Monte Carlo method on a normal personal computer. The focus of this article was not to optimize the 2-level Monte Carlo method but rather to use it as a comparison for the alternatively proposed GAM method. Complex evaluations, including dynamic transmission models, numerous intervention options, multiple countries, and considering a long time horizon, will become more common in the future. This in itself is an important reason for using GAM. But increasing the computational efficiency of complex models will also be helpful here. Third, although we use bootstrapping to provide a measure of uncertainty about the threshold value (and therefore avoid making assumptions of normality and homoscedasticity of the regression residuals), the nonparametric bootstrap itself has a limitation due to the nature of the statistic we are interested in. Due to sampling with replacement, it is possible that more or fewer parameter threshold values arise compared to the number obtained from the original PSA sample.

Parameter threshold analysis provides a useful and intuitively appealing source of information to inform policy makers and developers of technology. For example, threshold analysis can help to identify the maximum price that a government might be willing to pay for a drug. Such information can be used to inform research and development prior to drug licensing or price setting prior to marketing but also—and probably currently most frequently—to inform price negotiations when drugs (or other health care technology) are considered for reimbursement.^22^ We showed that this price could be under- or overestimated when based on deterministic threshold analysis. Furthermore, while EVPPI allows for the identification of uncertain input parameters that affect most on the optimal strategy,^2^ threshold analysis can single out more precisely at which values of an uncertain parameter the optimal strategy changes. This could inform the design of new trials to obtain more information about a particular uncertain parameter. Also, the threshold parameter value directly informs researchers and decision makers about the (change in) optimal strategy when a more precise estimate becomes available for a particular uncertain parameter based on new evidence. We believe that parameter threshold analysis has a wide range of applications, even beyond the field of health economics.

However, we recommend caution in instances where the parameter of interest is a noninfluential parameter (i.e., when it has a low EVPPI value). As shown in our example for $\mathrm{DO}I_{\mathrm{care}}$ in the setting of Uganda (WTP = $800, D=2), it is possible to obtain a threshold value for a noninfluential input parameter using GAM, but knowing the threshold value may have little consequence for policy makers, as the 95% CI covers almost the whole range of parameter values. In general, threshold values will be most relevant for uncertain input parameters that have an important impact on the optimal strategy of choice, and although GAM works well, it remains important to carefully interpret the results. If $B_{\mathrm{retain}}$ is low, we do not recommend the interpretation of the threshold value and the corresponding 95% CI due to the uncertainty.

In conclusion, we provide a flexible, easy to code, and fast alternative to the 2-level Monte Carlo approach for parameter threshold analysis. The GAM method provides correct estimates of parameter threshold value(s) when there is a nonlinear relationship between the uncertain input parameter of interest and the outcome of the health economic model. In this study, we only considered the threshold value for a single parameter. In the future, the GAM-based method could be extended to incorporate more than 1 parameter to conduct simultaneous multiparameter threshold analysis.

## Supplemental Material

Supplement_Probabilistic_Threshold_Analysis_20202525.rjf_online_supp – Supplemental material for A Computationally Efficient Method for Probabilistic Parameter Threshold Analysis for Health Economic Evaluations Click here for additional data file. Supplemental material, Supplement_Probabilistic_Threshold_Analysis_20202525.rjf_online_supp for A Computationally Efficient Method for Probabilistic Parameter Threshold Analysis for Health Economic Evaluations by Zoë Pieters, Mark Strong, Virginia E. Pitzer, Philippe Beutels and Joke Bilcke in Medical Decision Making
