# Supplementary material for: A Computationally Efficient Method for Probabilistic Parameter Threshold Analysis for Health Economic Evaluations
Source: Med Decis Making. 2020 Jul 5;40(5):669–79. doi: 10.1177/0272989X20937253 (PMC7401185; doi:10.1177/0272989X20937253)
Supplement: Supplement_Probabilistic_Threshold_Analysis_20202525.rjf_online_supp – Supplemental material for A Computationally Efficient Method for Probabilistic Parameter Threshold Analysis for Health Economic Evaluations [file Supplement_Probabilistic_Threshold_Analysis_20202525.rjf_online_supp.pdf]

A computationally-efficient method for probabilistic parameter threshold analysis for health economic evaluations.

Zoë Pieters (MSc)[1,2], Mark Strong (PhD)[3], Virginia E Pitzer (ScD)[4], Philippe Beutels (PhD)[2], Joke Bilcke (PhD)[2]

[1]I-BioStat, Data Science Institute, Hasselt University, Martelarenlaan 42, 3500 Hasselt, Belgium;
[2]Centre for Health Economics Research and Modeling Infectious Diseases (CHERMID), Vaccine and Infectious Disease Institute (VAXINFECTIO), University of Antwerp, Universiteitsplein 1, 2610 Wilrijk, Belgium;
[3]School of Health and Related Research (ScHARR), University of Sheffield, Sheffield, S1 4DA, UK;
[4]Department of Epidemiology of Microbial Diseases, Yale School of Public Health, Yale University,
P.O. Box 208034, 60 College St, New Haven, CT 0620-8034.

Appendix

A R code: A function to preform GAM and determine the parameter threshold value

```
#### Probabilistic Threshold analysis ####
      ## Obtaining the threshold value using GAM
      ## IMPORTANT: the default values are used in gam()

  # Input in the function:
      ## INB: a dataframe with the INB all D health care
          ## strategies (D is defined as all strategies -1,
          ## the latter being the baseline option)
          ## If D= 1 then INB is a vector
      ## baseline.name: a vector with the name of the baseline
          ## option to which all D health care strategies are
          ## compared to.
      ## parameter: a vector containing the values of the
```

```
            ## parameter of interest.
        ## parameter.name: an optional vector containing the
            ## name of the parameter for which the analysis
            ## is performed.
        ## INB and parameter are obtained from the PSA sample.
    # Return:
        ## threshold value: a list containing the parameter
            ## threshold values
        ## result: a list containing the results of GAM and
            ## the optimal strategy for each value of parameter.

pta<- function(INB, baseline.name, parameter, parameter.name=''){
  # Load the library for gam()
  library(mgcv)

  # Obtain values for use further downstream
  n<-length(parameter)

  if(class(INB)=='data.frame'){
    D <- ncol(INB)
  } else{
    D <- 1
  }

  # Sort the parameter values in ascending order
  sort_order <- order(parameter)
  sort_parameter<- parameter[sort_order]

  # 1) Fit a GAM
  if(D==1){
    # Use the appropriate settings in gam()
    result_gam<-gam(INB~s(parameter, bs='cr'))

    fitted_gam<-fitted(result_gam)

    # Sort the fitted values according to the input
    sort_fitted_gam<- fitted_gam[sort_order]
    colnames(sort_fitted_gam)<-colnames(INB)

  } else {
    fitted_gam<-data.frame(matrix(NA, nrow=n, ncol=D))

    for (i in 1:D){
    # Use the appropriate settings in gam()
```

```
      result_gam<-gam(INB[,i]~s(parameter, bs='cr'))

      fitted_gam[,i]<-fitted(result_gam)

    }

    # Sort the fitted values according to the input
    sort_fitted_gam<- fitted_gam[sort_order,]
    colnames(sort_fitted_gam)<-colnames(INB)
  }


# 2) Determine the parameter threshold value(s)
  ## When INB>0, then option d is cost-effective
  ## If INB<=0, the baseline option is the best choice.
  ## Create a vector of length n with only 0
  strategies<-cbind(rep(0, times=n), sort_fitted_gam)

  # Names of which alternative courses of actions.
  if(class(INB)=='data.frame'){
    colnames(strategies)<-c(baseline.name, colnames(INB))
  } else {
    colnames(strategies)<-c(baseline.name, 'alternative')
  }

  # For each value of parameter, which alternative course
    # of action has the highest expected INB.
  best_strategy<-colnames(strategies)[apply(strategies, 1, which.max)]

  result<-data.frame(sort_parameter, strategies, best_strategy)

  if(parameter.name!=''){
    colnames(result)[1]<-parameter.name
  }


  #Initialize vector that stores row numbers where
    # where change in optimal decision occurs
  rownr_result<-NULL
  for (i in 2:nrow(result)) {
    if(result$best_strategy[i]!=result$best_strategy[i-1]){
      rownr_result<-c(rownr_result,i)
    }
  }
```

```
  if(!is.null(rownr_result)){
    threshold<-data.frame(matrix(NA, nrow=length(rownr_result)+1,
                                 ncol=2))

    threshold[,1]<-c(NA, (result[rownr_result-1,1]+
                    result[rownr_result,1])/2)
    threshold[,2]<-c(as.character(result[c(rownr_result[1]-1),
                    ncol(result)]),
                    # Optimal strategy before threshold
                      as.character(result[rownr_result,ncol(result)]))
    colnames(threshold)<-c('TV', 'Strategy')
  } else {
    threshold<-paste('There are no threshold values.',
        result$best_strategy[1], 'is always the best.')
  }


  return(list(result=result, threshold=threshold))
}
```

B Samples values of θ_i in 2-level Monte Carlo approach

Table 1 displays the values that were used from the range of values of θ_i. For all settings, the same number of values were samples, $K = 7$.

Table 1: Values used for θ_i in the adjusted 2-level Monte Carlo approach (K=7)

Setting	Parameter	$\theta_i^{(k)}$
Nicaragua, WTP = \$1000, D=1	CFR_{hosp}	0.037, 0.038, 0.039, 0.040, 0.041, 0.042, 0.043
	$Pr(hosp)$	0.039, 0.040, 0.041, 0.042, 0.043, 0.044, 0.045
	DOI_{care}	10, 12, 14, 16, 18, 20, 22
Nicaragua, WTP = \$1000, $D = 2$	CFR_{hosp}	0.065, 0.066, 0.067, 0.068, 0.069, 0.070, 0.071
	$Pr(hosp)$	0.070, 0.071, 0.072, 0.073, 0.074, 0.075, 0.076
	DOI_{care}	10, 12, 14, 16, 18, 20, 22
Uganda, WTP = \$800, $D = 2$	CFR_{hosp}	0.055, 0.056, 0.057, 0.068, 0.069, 0.070, 0.071
	$Pr(hosp)$	0.058, 0.059, 0.060, 0.061, 0.072, 0.073, 0.074
	DOI_{care}	10, 12, 14, 16, 18, 20, 22
Cambodia, WTP = \$100, $D = 2$	CFR_{hosp}	0.052, 0.053, 0.054, 0.055, 0.079, 0.080, 0.081
	$Pr(hosp)$	0.057, 0.058, 0.059, 0.060, 0.082, 0.083, 0.084
Cambodia, WTP = \$8000, $D = 2$	CFR_{hosp}	0.001, 0.01, 0.03, 0.05, 0.07, 0.085, 0.1
	$Pr(hosp)$	0.01, 0.05, 0.09, 0.13, 0.17, 0.21, 0.25

Table 2: A comparison of the parameter threshold values, obtained with GAM, as reported in Table 2 in the main article with a cubic regression spline with dimension 10 and thin plate splines

		**GAM** Cubic regression splines, $L = 10$, $K = 10000$						**GAM** Thin plate regression splines, $K = 10000$					
θ_i	**EVPPI**	θ_i^*	$d^{(k^*+1)}$	Time[a]	95% CI	B_{retain}/B	Time[b]	θ_i^*	$d^{(k^*+1)}$	Time[a]	95% CI	B_{retain}/B	Time[b]
**Nicaragua, $\boldsymbol{WTP}$ = \$1000, 1 health care strategy ($\boldsymbol{D} = 1$; RC15) compared to no vaccination ($\boldsymbol{d_0}$)**													
CFR_{hosp}	700094	0.037	RC15	0.5	0.034-0.038	1000/1000	166.6	0.036	RC15	1.0	0.034-0.038	996/1000	340.3
$Pr(hosp)$	1276475	0.041	RC15	0.7	0.040-0.043	1000/1000	164.9	0.041	RC15	1.0	0.040-0.044	881/1000	334.6
DOI_{care}	0	None[c]	RC15	0.5	NA-NA	430/1000	165.1	None	RC15	1.0	NA-NA	577/1000	337.3
**Nicaragua, $\boldsymbol{WTP}$ = \$1000, 2 health care strategies ($\boldsymbol{D} = 2$; RC5 and RC15) compared to no vaccination ($\boldsymbol{d_0}$)**													
CFR_{hosp}	1860599	0.065	RC15	0.5	0.059-0.071	998/1000	175.4	0.064	RC15	1.5	0.058-0.074	990/1000	520.4
$Pr(hosp)$	2665148	0.070	RC15	0.5	0.065-0.074	1000/1000	176.8	0.070	RC15	1.5	0.065-0.074	826/1000	520.9
DOI_{care}	0	None	No vac	0.5	NA-NA	422/1000	182.1	None	No vac	1.4	NA-NA	434/1000	526.7
**Uganda, $\boldsymbol{WTP}$ = \$800, 2 health care strategies ($\boldsymbol{D} = 2$; RC5 and RC15) compared to no vaccination ($\boldsymbol{d_0}$)**													
CFR_{hosp}	18451230	0.058	RC5	0.7	0.053-0.061	998/1000	190.6	0.057	RC5	1.8	0.053-0.061	861/1000	559.6
		0.072	RC15		0.065-0.080			0.071	RC15		0.066-0.077		
$Pr(hosp)$	25018120	0.060	RC5	0.8	0.058-0.063	1000/1000	200.1	0.060	RC5	1.6	0.056-0.064	812/1000	556.2
		0.073	RC15		0.069-0.077			0.072	RC15		0.067-0.075		
DOI_{care}	4256	0.033	RC5	0.6	0.028-0.061	208/1000	194.1	0.033	RC5	1.7	0.028-0.061	332/1000	564.2
**Cambodia, $\boldsymbol{WTP}$ = \$100, 2 health care strategies ($\boldsymbol{D} = 2$; RC5 and RC15) compared to no vaccination ($\boldsymbol{d_0}$)**													
CFR_{hosp}	2788364	0.055	RC5	0.6	0.052-0.064	998/1000	188.6	0.055	RC5	1.5	0.049-0.060	994/1000	590.8
		0.079	RC15		0.075-0.089			0.083	RC15		0.078-0.092		
$Pr(hosp)$	4837512	0.060	RC5	0.8	0.058-0.062	1000/1000	191.3	0.060	RC5	1.8	0.058-0.064	972/1000	552.4
		0.085	RC15		0.081-0.090			0.084	RC15		0.081-0.088		
**Cambodia, $\boldsymbol{WTP}$ = \$8000, 2 health care strategies ($\boldsymbol{D} = 2$; RC5 and RC15) compared to no vaccination ($\boldsymbol{d_0}$)**													
CFR_{hosp}	0	None	RC15	0.9	NA-NA	975/1000	213.7	None	RC15	1.6	NA-NA	912/1000	562.5
$Pr(hosp)$	0	None	RC15	0.6	NA-NA	651/1000	187.8	0.001	RC15[d]	2.0	0.0006-0.003	305/1000	565.0

Notation: See Table 2 in the main article. [a,b] indicate the time needed to perform respectively the method and the bootstrap; [c] 'None' indicates that no parameter threshold value was obtained, meaning that the health care strategy with the highest expected INB remains the same and is denoted under $d^{(k^*+1)}$; [d] the optimal strategy before the threshold value was RC5 instead of no vac.

C Sensitivity Analysis

In Table 2, we compare the threshold values obtained via different smoothing option in the `gam()` function. In Table 2 of the main article, we used cubic regression splines with dimension 20. In Table 2, we use again cubic regression splines, but now with dimension 10 (default option in R [1]). In addition, we use thin plate regression splines to obtain the threshold value. The advantage of using thin plate regression splines is that it is not necessary to specify the number of knots (and thus the dimension), i.e. it is knot free [2].

D Bootstrap procedure for non-influential parameters

In this section, we elaborate on the observation of low B_{retain} for non-influential parameters. For comparison, we again explore the results for the non-influential parameter DOI_{care}. In addition, we provide results for the proportion of typhoid cases infected with an antimicrobial resistant (AMR) strain (AMR), the burden of AMR cases relative to AMR sensitive cases ($BurdenAMR$), and the relative duration illness for patients not seeking medical care ($DOI_{nocare/care}$). The characteristics of each input parameter are shown in Table 3.

Table 3: Distributional characteristics of the non-influential input parameters.

**Parameter**	**Mean**	**Median**	**95% CI**[a]	**Uncertainty distribution**
AMR (%)	0.5	0.5	0.02-0.98	$Uniform(0,1)$
$BurdenAMR$	2	2	1-3	$Uniform(1,3)$
$DOI_{nocare/care}$	0.5	0.5	0.02-0.98	$Uniform(0,1)$

[a] 95% credible. interval

In the case of non-influential parameters (such as DOI_{care} in Table 2 of the main article), the bootstrap samples retained are much lower compared to influential parameters. Remember that a non-influential parameter will not affect the optimal decision, and hence there is no threshold value. If B_{retain} is low, then in the majority of the bootstrap samples, at least one threshold value is obtained.

We compared the results from different non-influential input parameters. Table 4 displays the results from a GAM using cubic splines regression ($L = 20$, $K = 10000$). We observe that $EVPPI = 0$ defines non-influential parameters. Defining a parameter as non-influential based on a low EVPPI value is very arbitrary since for $EVPPI > 0$ parameter threshold value(s) are observed (See also Table 2 in the article, DOI_{care} for Uganda ($WTP =$

800\$, $D = 2$)). The 95% CI surrounding the threshold value is usually very wide, rendering the interpretation of the threshold value invaluable. The reason for observing at least one threshold value in the case of non-influential parameters is that (1) the shape of the GAM is affected by the individual bootstrap samples and (2) the uncertainty surrounding the health care strategy due to other parameters might determine greatly whether the health care strategy come forward as the most optimal strategy or not. Therefore, we do not recommend to interpret threshold values and corresponding 95%CI when B_{retain} is low.

Table 4: Results from GAM for non-influential parameters

		**GAM** Cubic regression splines, $L = 20$, $K = 10000$					
θ_i	**EVPPI**	θ_i^*	$d^{(k^*+1)}$	Time[a]	95% CI	B_{retain}/B	Time[b]
**Nicaragua, WTP = \$1000, 1 health care strategy ($D = 1$; RC15) compared to no vaccination (d_0)**							
AMR	0	None	RC15	0.7	NA-NA	675/1000	185.8
$BurdenAMR$	65	1.010	RC15	0.6	1.004-1.213	419/1000	183.8
$DOI_{nocare/care}$	0	None	RC15	0.5	NA-NA	843/1000	184.0
**Nicaragua, WTP = \$1000, 2 health care strategies ($D = 2$; RC5 and RC15) compared to no vaccination (d_0)**							
AMR	194461	0.667	RC15	0.6	0.514-0.808	192/1000	243.5
$BurdenAMR$	390545	2.230	RC15	1.0	1.863-2.589	614/1000	238.5
$DOI_{nocare/care}$	0	None	No vac	0.7	NA-NA	77/1000	202.5
**Uganda, WTP = \$800, 2 health care strategies ($D = 2$; RC5 and RC15) compared to no vaccination (d_0)**							
AMR	1375066	0.076	No vac[d]	0.6	0.009-0.474	146/1000	204.9
		0.463	RC5		0.325-0.772		
		0.779	RC15		0.585-0.978		
$BurdenAMR$	3255705	1.827	RC5	0.6	1.567-2.066	208/1000	202.2
		2.440	RC15		2.262-2.665		
$DOI_{nocare/care}$	39382	0.806	No vac[d]	0.6	0.155-0.844	18/1000	202.8
		0.892	RC5		0.279-0.966		

Notation: See Table 2 in the main article. [a,b] indicate the time needed to perform respectively the method and the bootstrap; [c] 'None' indicates that no parameter threshold value was obtained, meaning that the health care strategy with the highest expected INB remains the same and is denoted under $d^{(k^*+1)}$; [d] the optimal strategy before the threshold value was RC5.

Bibliography

[1] R Core Team. R: A Language and Environment for Statistical Computing. Vienna, Austria: R Foundation for Statistical Computing; 2018. Available from: `https://www.r-project.org/`.

[2] Wood SN. Generalized Additive Models: An introduction with R. 1st ed. New York: Chapman and Hall/CRC; 2006.
